# Simulation and experimental verification of the precision finishing method for optical free-form surface segmentation

**DOI:** 10.1371/journal.pone.0314489

**Published:** 2025-02-21

**Authors:** Chenhua Jiang, Enzhong Zhang, Wei Zhang, Jiaqi Hu, Jiechen Guo, Xiaodong Li

**Affiliations:** School of Mechatronical Engineering, Changchun University of Technology, Changchun, China; Julius-Maximilians-Universitat Wurzburg, GERMANY

## Abstract

Optical free-form surfaces are often used to manufacture optical components such as lenses and mirrors, and free zone surface optical components are widely used in aerospace optics. To address the issues of low processing quality and overall efficiency in the machining of aluminum alloy free-form surfaces, an efficient numerical control machining method based on surface segmentation has been proposed. The segmentation of free-form surfaces is divided into two stages: the recognition of surface partitions and the determination of surface boundaries. Initially, the free-form surface is roughly classified into three types of regions: convex (planar), concave, and saddle-shaped, based on its curvature characteristics. The surface is then further segmented using the fuzzy c-means clustering algorithm. Subsequently, the Voronoi diagram algorithm is employed to construct the boundaries of the free-form surface ultimately. In a UG simulation, the segmentation machining method was compared with the conventional overall machining method, demonstrating a reduction in the machining path by 12.18% and machining time by 13.92%. Finally, experiments revealed a 12.28% reduction in machining path length and a 12.56% reduction in machining time using the segmentation machining method. This verifies the practicality of the free-form surface segmentation approach and proves that it can effectively enhance machining efficiency and quality.

## 1. Introduction

With the development of industrialization, there is an increasing expectation for high-standard results in product design and production. Complex surface geometries can no longer rely on traditional parameterized surface descriptions, which have been replaced by more advanced free-form surfaces [1, 2]. In the engineering field, free-form surfaces are a frequently encountered and structurally complex type of surface that finds widespread application in various industries such as shipbuilding, aerospace, and automotive manufacturing [3]. Modern products require parts with increasingly complex shapes and higher precision. To ensure the machining quality and efficiency of these large components, the surfaces to be processed are divided into several different regions, and different machining methods are designed for each region. Additionally, appropriate settings for processing parameters and paths are established for each area to optimize the machining process.

The processing of free-form surfaces using a single machining method is likely to result in low processing efficiency and inferior overall quality. Therefore, segmented machining is necessary. The process of segmented machining for free-form surfaces generally consists of three stages: rough machining, precision machining, and reference machining [4]. In the rough machining stage, the main goal is to remove material, with high cutting forces and low requirements for surface quality. In the finishing stage, the material removal rate is low, the cutting forces are smaller, and there are higher requirements for surface quality. In the finishing stage, the residual height left from the finishing process is removed through grinding and polishing.

This paper proposes a method for the segmented processing of free-form surfaces and validates it through simulation and experimental testing. The surface treatment can be divided into two steps: 1. Surface partition recognition, 2. Determination of surface boundaries. The free-form surface is roughly divided into three categories: convex (planar), concave, and saddle-shaped areas, based on the surface curvature characteristics. Then, the fuzzy c-means clustering algorithm is used for clustering calculations to achieve the subdivision of the free-form surface. Based on the results of the clustering calculations, the Voronoi diagram algorithm is ultimately used to construct the boundaries of the free-form surface. By comparing the three-axis numerical control (NC) segmented milling simulation with the overall milling simulation in UG, it is found that the segmented processing method reduces the machining path length by 12.28% and the machining time by 12.56%. Finally, the effectiveness of the segmented processing method is verified through experimental testing, which can effectively improve the surface machining quality and efficiency.

## 2 Related work

Currently, there has been extensive research on the segmentation of specific free-form surfaces by many scholars. Yazui Liu [5] was the first to adopt T-spline curve technology for the segmentation of free-form surfaces, overcoming two limitations in current research on free-form surface processing: (1) the fixed rectangular topology of NURBS leads to additional sampling points and increased computational effort due to global sampling; (2) the edges of the segmented regions are typically represented by analytical or discrete points, resulting in possible gaps in the free-form surface at the edge regions when used as a trimming surface. Leveraging the flexibility of T-spline surface structures and sampling by surface, an innovative segmentation technique based on T-spline surfaces has been proposed, which significantly improves cutting efficiency. Additionally, by analyzing clustering parameters, a method based on curvature is selected to subdivide the surface and extract isolated features. The boundaries of the segmented regions are defined using T-spline local refining and sliding box technology, while a rectangular grid ensures the continuity of the boundaries and eliminates gaps between regions. Tools of different sizes are used to generate tool paths for the partitioning, thereby reducing path length.

Zezhong C. Chen [6] proposed a method for automatic surface segmentation and path generation. This method divides a complex free-form surface into several sub-surfaces based on differences in their geometric characteristics. Then, according to the machining characteristics of each sub-surface, the method automatically generates five-axis CNC tool paths for each sub-surface. Liu and colleagues [7–9] proposed a domain-based machining method based on surface curvature. This method divides the surface according to the surface curvature and defines the center based on a clustering algorithm, followed by segmented simulation and machining.

Roman [10] employed the fuzzy c-means method for the segmentation of free-form surfaces, with the boundaries of each surface patch delineated using the nearest neighbor method in the u-v plane. Furthermore, the k-means clustering method was used to subdivide the surface into patches, and the boundaries between patches were identified using the minimum within-class distance method. This approach may result in several patches that exceed the count of concave, convex, and saddle regions on the surface, which could increase the processing time after segmentation.

Bezbarush [11] introduced a free-form surface machining method based on the principle of normal curvature. This method involves decomposing the selected surface into multiple surfaces with the maximum convex curvature and smoothness. Primary tool paths are generated in a specified region, and secondary tool paths are obtained within the same region using the residual height method. Experimental results have demonstrated that the primary tool paths significantly reduce machining time in the direction of the maximum convex curvature, and the tool paths also reduce the data file size in the direction of maximum smoothness. Elber and Cohen [12] adopted a hybrid technique that combines symbolic and numerical operations to divide free-form surfaces into convex, concave, and saddle-shaped regions, and defined the global limits of surface curvature. Bui Ngoc Tuyen [13] proposed a new segmentation method based on Gaussian and mean curvature to subdivide free-form surfaces, utilizing the Freeman algorithm to delineate the boundaries of local surface patches, and verified this method experimentally with B-spline surfaces.

After determining the distribution of individual patches on the surface, the definition of boundaries between these patches is of crucial importance for subsequent processing. Liu and colleagues [14] proposed a tool path method for processing on a five-axis machine tool, which utilizes the k-means clustering algorithm to divide the free-form surface into regions and then defines the boundaries between these regions using B-spline curves. Mao utilized the K-nearest neighbor (KNN) algorithm based on K-d trees to search for discrete points on a surface, resulting in the formation of several point clusters. By linearly connecting the most exterior points of each discrete point cluster, the algorithm defines the boundary lines of the surface patches, thereby dividing the surface into distinct regions.

Lamikiz and Tsai [15, 16], along with other researchers, have developed a general mathematical model using the D-H (Denavit-Hartenberg) method, which accounts for various factors including the basic structure of the machine tool, types of joints, and tool configurations. This model provides an essential reference for the kinematic modeling and analysis of five-axis CNC (Computer Numerical Control) machines. More complex five-axis machines, which may include up to three rotary axes, pose challenges for traditional kinematic analysis. To address this, Yun and colleagues [17] have proposed a reliable, non-numerical inverse kinematic analysis method suitable for both two-rotary-axis and three-rotary-axis CNC systems. Hong and colleagues [18] have analyzed and summarized all potential error scenarios in five-axis synchronous machining, elucidating the impact of various error motions on machining precision. They have modeled these complex errors, simplifying them into fundamental geometric errors related to the position of the rotary axes. Sneha and colleagues [19] have introduced a novel approach to error handling, inserting quintic spline boundary micro-samples at the beginning and end of adjacent tool path segments to ensure C3 continuity at the connection points and to smooth the local tool path. Currently, workpiece coordinate transformation offers a novel solution to this issue, with Li and colleagues [20] establishing a new geometric error model that identifies errors in five-axis machines through an additional workpiece coordinate transformation. This enables preemptive error handling to minimize interference from errors.

In summary, based on the fuzzy c-means clustering algorithm, this paper combines the Voronoi diagram algorithm for slice planning of complex surfaces. Different machining methods are adopted for machining according to the differences in surface curvature after segmentation.

## 3 Point cloud definition and acquisition

### 3.1 Definition of point cloud data

**Fig 1:**
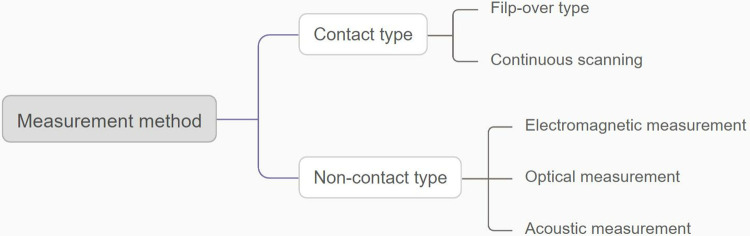
Measurement method of point cloud data.

A point cloud is a collection of many independent points distributed in three dimensions, where each point precisely corresponds to a specific sampling location on the surface of a physical object. Using accurate 3D measurement techniques such as laser scanning, optical measurement or tactile probes, data from these points is collected and recorded to form a detailed digital representation of the object’s surface. Each point in a point cloud contains a wealth of information, the most basic of which are its three-dimensional coordinates (X, Y, Z axis position information) and RGB color values. Three-dimensional coordinates ensure the spatial positioning accuracy of points, while RGB color information provides additional reference dimensions for subsequent data visualisation, analysis and processing, making data points more intuitive and conducive to effective identification and classification in complex data processing processes. The specific method for obtaining point cloud data is shown in Fig 1.

Compare and analyse the advantages and disadvantages of the contact and non-contact instruments mentioned in Fig 1, the results are shown in Table 1.

**Table 1:** Contact measuring instrument and non-contact measuring instrument.

	Contact measurement	Non-contact measurement
**advantage**	High measurement accuracy	Fast measurement speed
	Low requirements on workpiece surface roughness	No radius compensation
	Direct measurement of the geometric features of the surface	Measurement of inaccessible parts
	The impact of environmental interference is small	Surface accuracy is not affected
**shortcoming**	Small surfaces cannot be measured	The measurement accuracy is low
	The measuring head needs radius compensation	Steepness cannot be measured
	The probe is prone to wear	Special geometries cannot be measured
	Slow measurement speed	The workpiece surface quality has great influence on the measurement accuracy

### 3.2 Point cloud data acquisition

In order to obtain the point cloud data of the original workpiece, an in-house developed coordinate measuring machine was used. This measuring machine possesses precise measurement capabilities and a wide range of measurement, with the measurement range of each axis detailed in Table 2:

**Table 2:** CMM measuring range.

	Measuring range (mm)
X-axis	0–270
Y-axis	0–245
Z-axis	0–225

The range design ensures that the measuring machine can adapt to the measurement requirements of different workpiece sizes. Keyence’s GT2-H50 contact sensor is used in the measuring head section of the gauge. The sensor is known for its high precision and high resolution, with a measurement accuracy of 3.5μm and a resolution of 0.5μm, ensuring the accuracy and fineness of the point cloud data.

**Fig 2:**
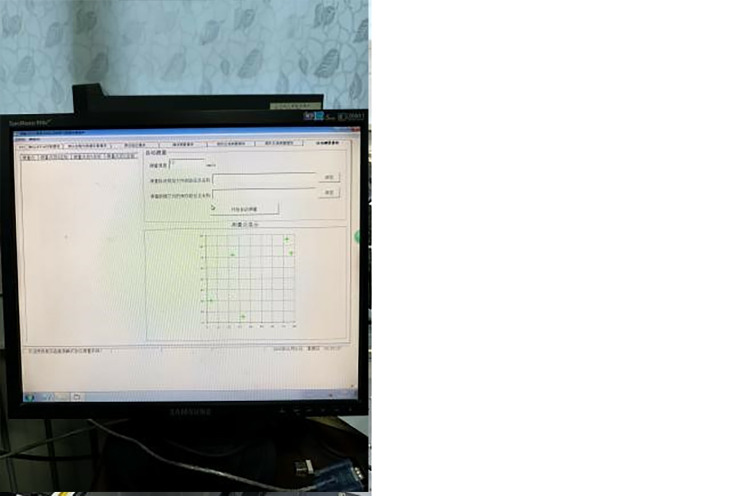
Measurement process diagram of CMM.

In addition, the design and functions of the measuring device, control system and data display device of the CMM are also shown in detail in Fig 2. The measuring machine has a friendly user interface, easy operation, real-time display of measurement data and progress, and provides users with an efficient and intuitive measurement environment. Together, these features form a fully functional metrology solution that meets the need for accurate measurement in industrial applications.

The steps of using CMM to obtain surface point cloud data mainly include

**Fig 3:**
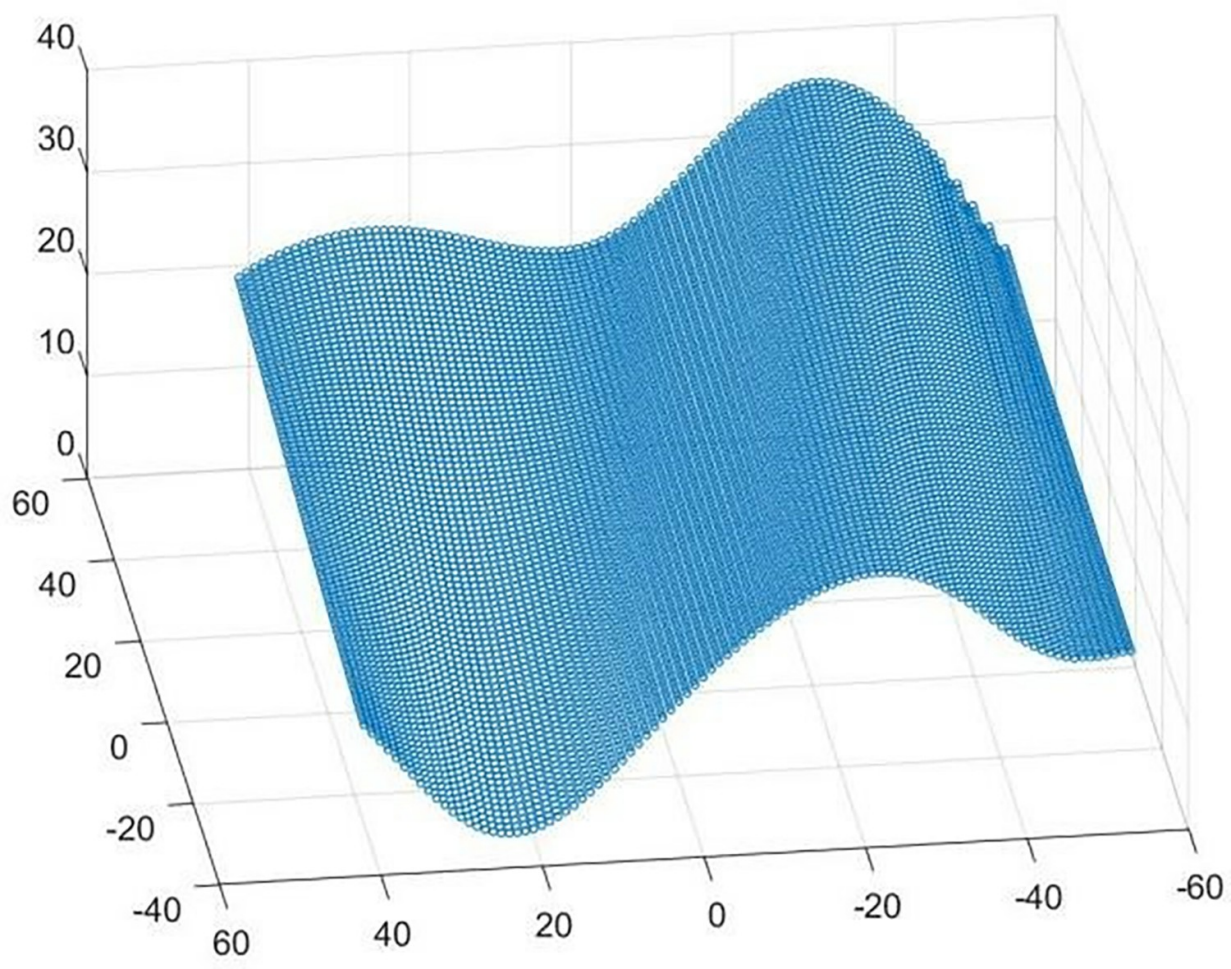
Point cloud data.

1. Selection & Compatibility of Workpieces: When selecting a workpiece for measurement, it is necessary to ensure that the size and shape of the workpiece are suitable for the working range of the CMM. In particular, it is necessary to select parts that allow the probe to effectively cover the peaks and valleys of the free-form surface within its working range, ensuring that the accuracy of the data is not lost when measuring beyond the Z-axis measurement capability.
2. The importance of fixture positioning: In the measurement process, it is important to ensure correct clamping and accurate positioning of the surface under test. For free-form surfaces with a regular cuboid bottom surface, although the clamping process is relatively simple, it is still necessary to ensure that the probe can move along the set trajectory without obstruction and accurately establish the coordinate system.
3. Establishment of Coordinate System: Establishing a unified coordinate system is essential to simplify subsequent data processing. For regular quadrilateral free-form surfaces, one of the angles can be selected as the coordinate origin to unify all data processing.
4. Measurement path strategy: A well-designed measurement path is essential to obtain high-quality point cloud data. In contact measurement, although dense data points can improve the reconstruction accuracy, it also increase the measurement time and data processing. Therefore, the measurement path of the sheet is adopted, and the measurement efficiency is optimised while data quality is guaranteed by setting a reasonable path and measurement point interval (both of which are 1mm).
5. Data preservation and processing: The measured point cloud data should be saved in a text file (.txt) format. This not only facilitates the storage and transmission of data, but also simplifies the steps of data processing. After completing the above steps, the detailed point cloud data of the free-form surface is obtained, and its visualization results are shown in Fig 3, which provides an important basis for subsequent analysis and application.

## 4 The definition of free-form surfaces and surface geometric shapes

### 4.1 Free-form surface representation

In general, the design of free-form surfaces is completed directly on a computer through control points. Common design methods include Bezier surfaces, B-spline surfaces, and Non-Uniform Rational B-spline (NURBS) surfaces. These surfaces have become the mainstream method for defining free-form surfaces in today’s CAD/CAM systems. Particularly, NURBS is widely used due to its excellent intuitiveness and advantages in local processing, convergence, and approximation capabilities, making it the most commonly employed curve fitting technique currently. For these reasons, this study chooses NURBS to define free-form surfaces.

There are three equivalent expressions for the NURBS surface equation, including homogeneous coordinate representation, rational basis function representation, and rational fraction representation. In this paper, the rational fraction representation is adopted to describe NURBS surfaces.

Specifically, a *k×l*-degree NURBS surface in space can be represented by a piecewise rational polynomial function:

$$p(u,v)=\frac{\sum_{i=0}^{n}\sum_{j=0}^{m}w_{i,j}d_{i,j}N_{i,k}(u)N_{j,l}(v)}{\sum_{i=0}^{n}\sum_{j=0}^{m}w_{i,j}N_{i,k}(u)N_{j,l}(v)}$$ (1)

**Fig 4:**
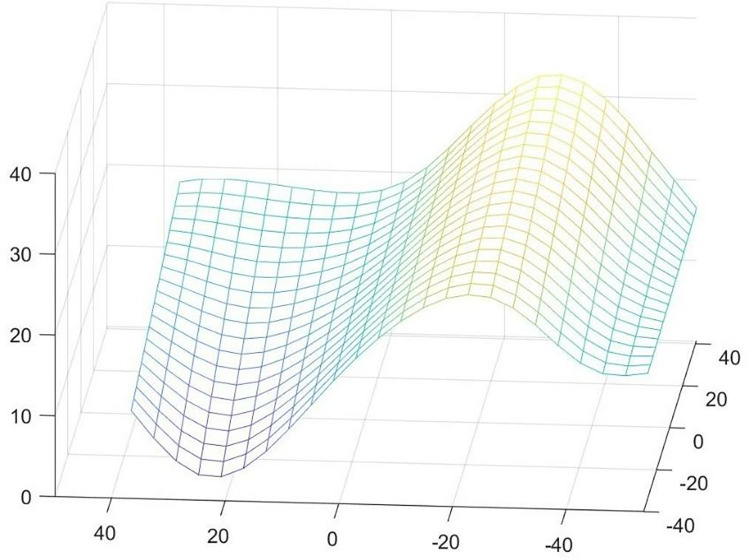
3D fitting diagram of free-form surfaces.

In the equation: *P*(*u*,*v*) is the parameter equation of the free-form surface *S*;

Where: di,j (where i = 0,1,…,n; j = 0,1,…,m) are the control points forming a bidirectional control grid; w_i,j_ are the weight factors of the NURBS surface, associated with the control points d_i,j_, and are defined such that w_0,0_, w_n,0_, w_0,m_, and w_n,m_ are all greater than 0. The remaining weight factors w_i,j_ are non-negative and ensure that no adjacent sequence of k×l weight factors is zero. u and v are the parameter variables in the two directions of the NURBS surface; N_i,k_(u) (where i = 0,1,…,n) and N_i,l_(v) (where j = 0,1,…,m) are the non-rational B-spline basis functions of degree k in the u-direction and degree l in the v-direction, respectively, determined by the node vectors U = [u_0_, u_1_, u_n+k+1_] and V = [v_0_, v_1_, v_m+1+1_] following the de Boor-Cox recursion formula. When determining the node vectors U and V, the domain they define is a standard unit square, and they are divided into (n−k+1)(m−l+1) rectangle elements by the interior node lines, such that the parameter range is 0<u,v<1. An example of the free-form surface studied in this paper is shown in Fig 4.

### 4.2 Surface geometry shape

Surfaces can be represented in implicit, explicit, or parametric forms. The parametric form of a spatial surface is as follows:

$$S(u,v)=(\begin{array}{l} S_{x}(u,v)\\ S_{y}(u,v)\\ S_{z}(u,v) \end{array})$$ (2)

The following sections will introduce several geometric properties of surfaces.

**1) Unit normal vector.** The unit normal vector at a point (*u*,*v*) on the surface *S*(*u*,*v*) can be calculated as follows:

$$n(u,v)=\frac{\left(S_{u}\times S_{v}\right)}{\left|S_{u}\times S_{v}\right|}$$ (3)

where *S*_*u*_ and *S*_*v*_ are the surface tangent vectors along the parameter directions *u* and *v*.

**2) Gaussian curvature (K) and mean curvature (H).** The Gaussian curvature K of a point *P*(*x*,*y*,*z*) on the surface *S*(*u*,*v*) is given by the following formula:

$$K=\frac{LN-M^{2}}{EG-N^{2}}$$ (4)

where:

$$E=\frac{\partial S}{\partial u}\frac{\partial S}{\partial u};F=\frac{\partial S}{\partial u}\frac{\partial S}{\partial v};G=\frac{\partial S}{\partial v}\frac{\partial S}{\partial v}$$ (5)

$$L=n\frac{\partial^{2}S}{\partial u^{2}};M=n\frac{\partial^{2}S}{\partial u\partial u};N=n\frac{\partial^{2}S}{\partial v^{2}}$$ (6)

The mean curvature H is defined as:

$$H=\frac{1}{2}(\frac{EN-2FN+GL}{EG-F^{2}})$$ (7)

The principal curvatures are given by the following expressions:

$$K_{\max}=H+\sqrt{H^{2}-K}$$ (8)

$$K_{\min}=H-\sqrt{H^{2}-K}$$ (9)

Also Gaussian curvature and mean curvature need to satisfy:

$$H^{2}\geq K$$ (10)

Based on the values of the Gaussian curvature K and the mean curvature H, the local surface shape around a point can be classified into four distinct types [1, 10]:

1. K ≥ 0 and H < 0: The local surface shape is convex.
2. K ≥ 0 and H > 0: The local surface shape is concave.
3. K < 0 and H < 0 or H > 0: The local surface shape is saddle-shaped.
4. K = 0, H = 0: The local surface shape is planar.

Note that the case where K > 0 and H = 0 does not exist, as the Gaussian curvature and the mean curvature must satisfy the condition *H*^2^≥*K*.

The signs of the Gaussian curvature K and the mean curvature H at a point on a surface can determine the type and local surface shape of the point, as shown in Table 3).

**Table 3:** The relationship between the surface shape and the Gaussian curvature (K) and the mean curvature (H).

Gaussian curvature (K)	Mean curvature (H)	Local surface shape
K≥0	H<0	Convex area
K≥0	H>0	Concave area
K<0	H<0	Saddle area
K<0	H>0	Saddle area
K = 0	H = 0	Flat area

## 5 The division of free surfaces and the definition of boundaries

### 5.1 The initial division of the surface

This paper aims to facilitate more effective processing and analysis through the segmentation of point cloud data. Segmentation algorithms based on surface curvature are a widely employed technique, which utilizes the curvature information of each point within the point cloud to achieve data segmentation. This method can effectively partition the point cloud into multiple regions, facilitating deeper analysis and subsequent processing. Moreover, the application of curvature information includes the extraction of surface edges, corners, and other features, as well as surface fitting and feature extraction. In practical operations, appropriate curvature thresholds and processing strategies can be selected based on specific requirements to achieve the best results.

**Fig 5:**
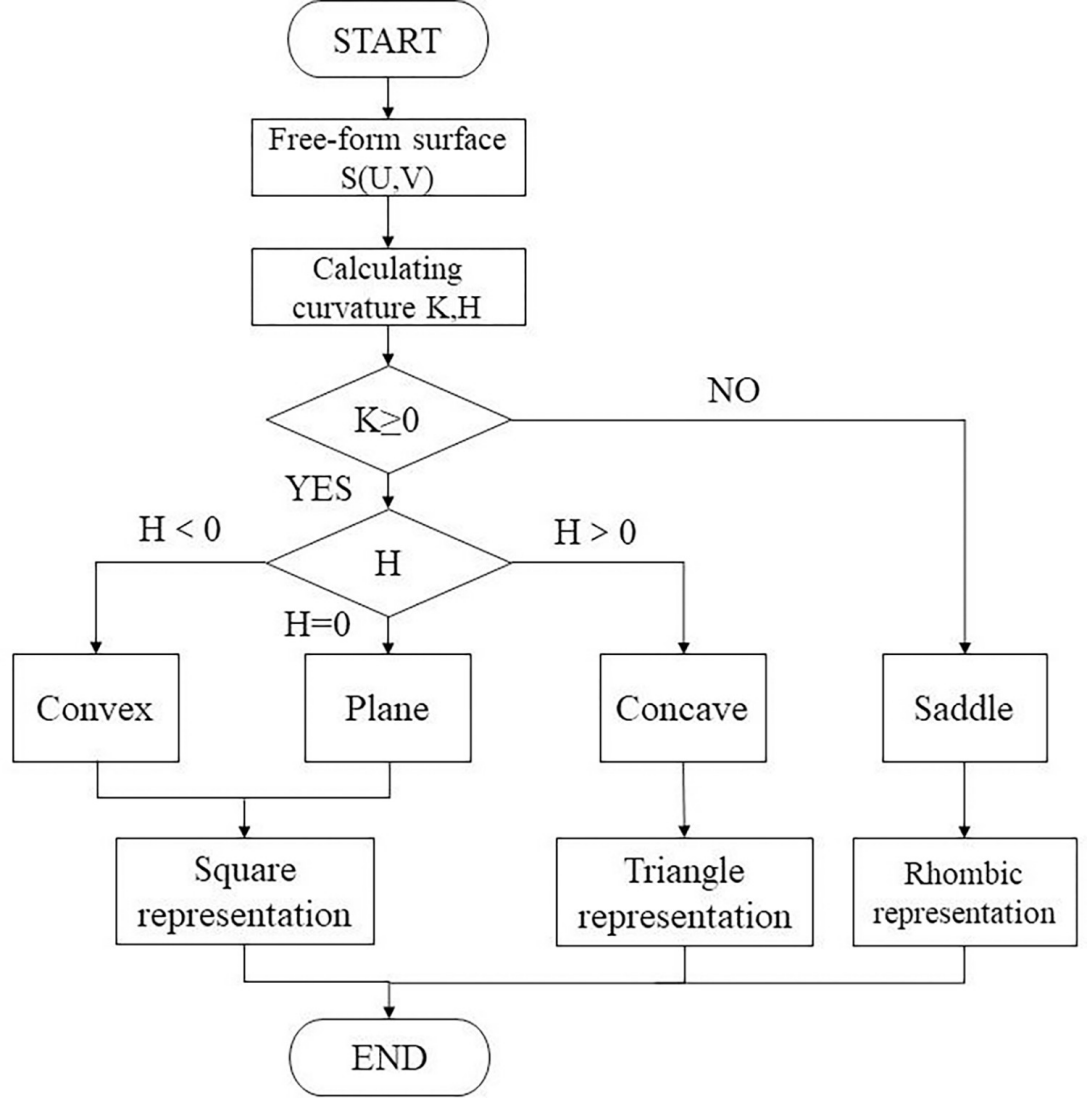
Flow chart of rough surface.

**Fig 6:**
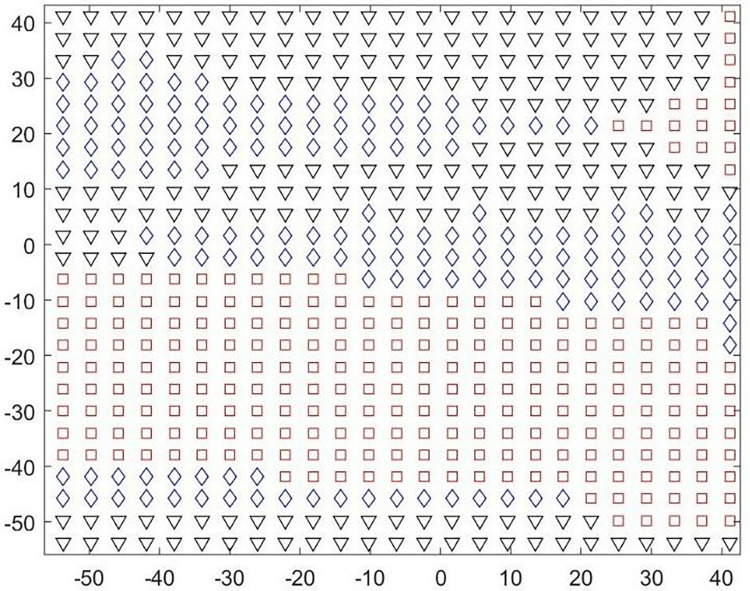
Initial division diagram based on curvature.

In this study, the surface is divided into convex regions (including planar regions), concave regions, and saddle regions based on the Gaussian curvature K and the mean curvature H, and are represented by the symbols □, ◇, ▽, respectively. Due to the visual continuity of the convex regions and the planar regions, we treat them as a whole, which not only reduces the number of segmented regions but also improves processing efficiency. Figs 5 and 6 are flowcharts and preliminary division diagrams of the surface initial division, respectively.

### 5.2 Surface subdivision

**Fig 7:**
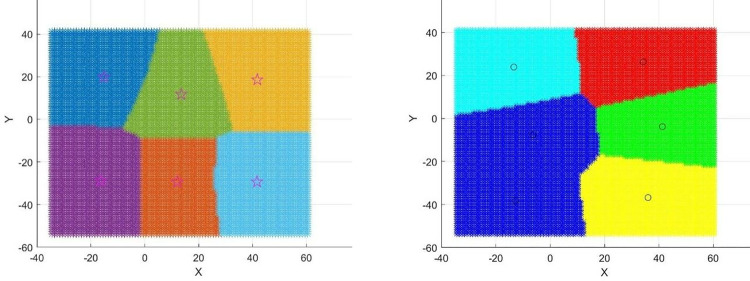
Comparison of FCM and K-means clustering effects under the same number of clusters: a. K-means Algorithm; b. FCM Algorithm.

Using the previously described method, the surface has been effectively classified into convex regions (including planar regions), concave regions, and saddle regions. To further refine the segmentation of the surface, this study employs the Fuzzy C-Means clustering method (FCM). Although there are various techniques for surface segmentation, such as the K-means algorithm and the FCM algorithm, Fig 7 shows the division diagram of FCM and K-means clustering effect surfaces under the same number of clusters. The FCM clustering algorithm stands out for its more accurate classification results in line with real-world situations and its ability to provide a relatively optimal solution. The algorithm optimizes details through multiple iterative processes, resulting in a more refined data segmentation and higher clustering accuracy, hence its selection for this study.

After the above processing, the free surface has been roughly segmented into three types of regions: convex (planar), concave, and saddle pieces. Subsequently, the Fuzzy C-Means clustering method (FCM) is employed to further segment the surface.

After the above processing, the free-form surface has been coarsely divided into three types of area slices: convex (planar), concave, and saddle piece surfaces. Then the surface is further divided by the fuzzy C-means clustering method FCM (Fuzzy C-Means).

Fuzzy clustering is one of the important research branches in many fields, including knowledge discovery and pattern recognition. As the scope of research expands, both in scientific research and practical applications, there is an increasing demand for higher standards of clustering results from various perspectives. Fuzzy C-Means clustering (FCM) is currently a popular clustering method. This method utilizes the concept of geometric proximity of data points in Euclidean space, allocating these data to different clusters and then determining the distances between these clusters. The Fuzzy C-Means clustering algorithm has laid the foundation for other fuzzy clustering analysis methods both theoretically and practically, and is the most widely used. The Fuzzy C-Means clustering (FCM) method is as follows:

Let n data samples be represented as X = {*x*_1_,*x*_2_,…,*x*_*n*_}, with c (where 2 ≤ c ≤ n) being the number of types into which the data samples are to be divided. The corresponding c categories are represented by {A1, A2,…, Ac}, and U is the similarity classification matrix. The centroid of each category is {v1, v2,…, vc}, and μk(xi) is the membership degree of data sample xi to category Ak (abbreviated as μik). The objective function J_b_ can be expressed as follows:

$$J_{b}(U,v)=\overset{n}{\underset{i=1}{\sum }}\overset{c}{\underset{k=1}{\sum }}{\left(\mu_{\mathrm{ik}}\right)}^{b}{\left(d_{\mathrm{ik}}\right)}^{2}$$ (11)

Where $d_{ik}=d(x_{i}-v_{k})=\sqrt{\sum_{j=1}^{m}{\left(x_{ij}-v_{kj}\right)}^{2}}$. dik is the Euclidean distance used to measure the distance between the i-th sample xi and the centroid of the k-th category; m is the number of features in the sample; b is the weighted parameter with a range of 1 ≤ b ≤ ∞. The Fuzzy C-Means clustering method involves seeking the best classification that minimizes the function value jb. It requires that the sum of the membership degrees of a sample to all clusters equals 1, i.e., it satisfies:

$$\sum_{j=1}^{c}\mu_{j}(x_{i})=1,\;i=1,2,\cdots ,n$$ (12)

Eqs (3) and (4) are used to calculate the membership degree μik of sample x_i_ to category A_k_ and the c cluster centroids {v_i_}, respectively:

$$\mu_{ik}=\frac{1}{\sum_{j=1}^{c}{\left(\frac{d_{ik}}{d_{jk}}\right)}^{\frac{2}{b-1}}}$$ (13)

Define I_k_ = {i | 2 ≤ c ≤ n; d_ik_ = 0}, for all i of category, i ∈ I_k_, μ_ik_ = 0.


$$v_{ij}=\frac{\sum_{k=1}^{n}{\left(\mu_{ik}\right)}^{b}x_{kj}}{\sum_{k=1}^{n}{\left(\mu_{ik}\right)}^{b}}$$ (14)

**Fig 8:**
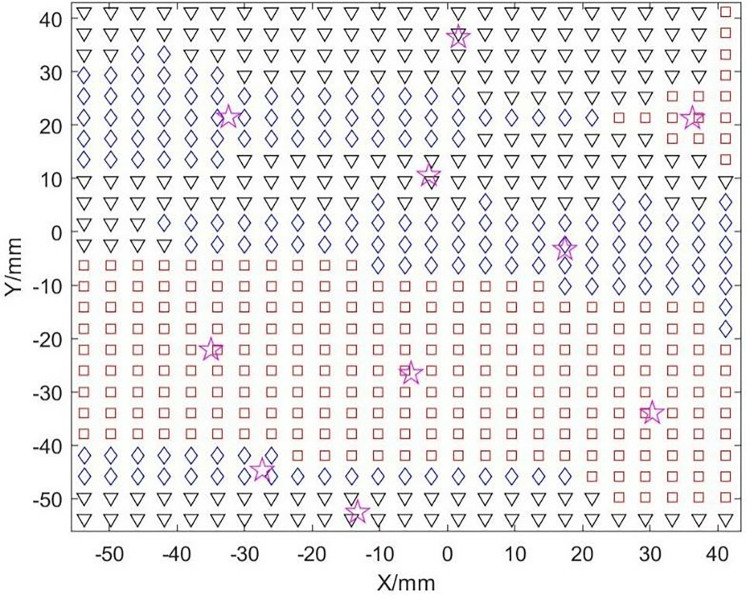
Cluster centroids and subdivided regions.

By iteratively modifying the cluster centroids, data membership, and performing classification using Eqs (3) and (4), when the algorithm converges, theoretically, the cluster centroids of each category and the membership degrees of each sample to different pattern classes are obtained, thus completing the fuzzy clustering partition. Fig 8 shows the cluster centroids and subdivision regions. Although FCM has a high search speed, it is a local search algorithm and is very sensitive to the initial values of the cluster centroids. If the initial values are chosen incorrectly, it may converge to a local minimum.

### 5.3 Surface boundary definition

**Fig 9:**
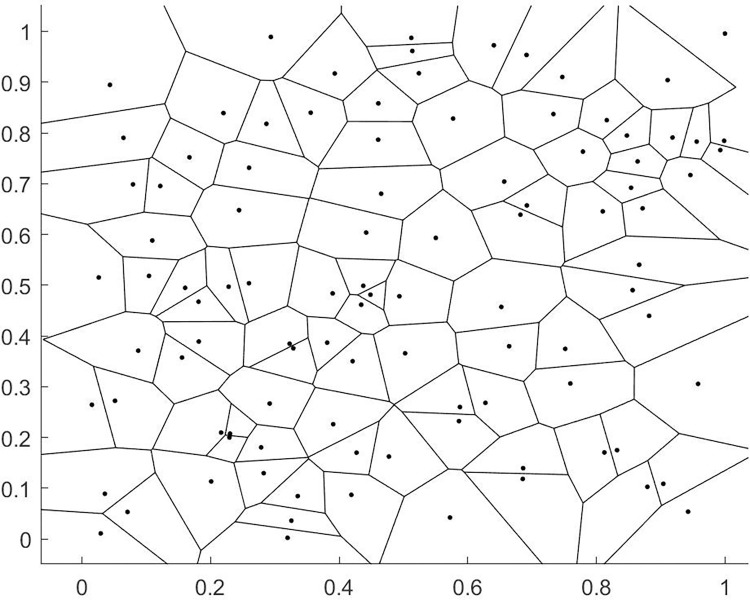
Voronoi diagram of a region with 100 stations.

**Fig 10:**
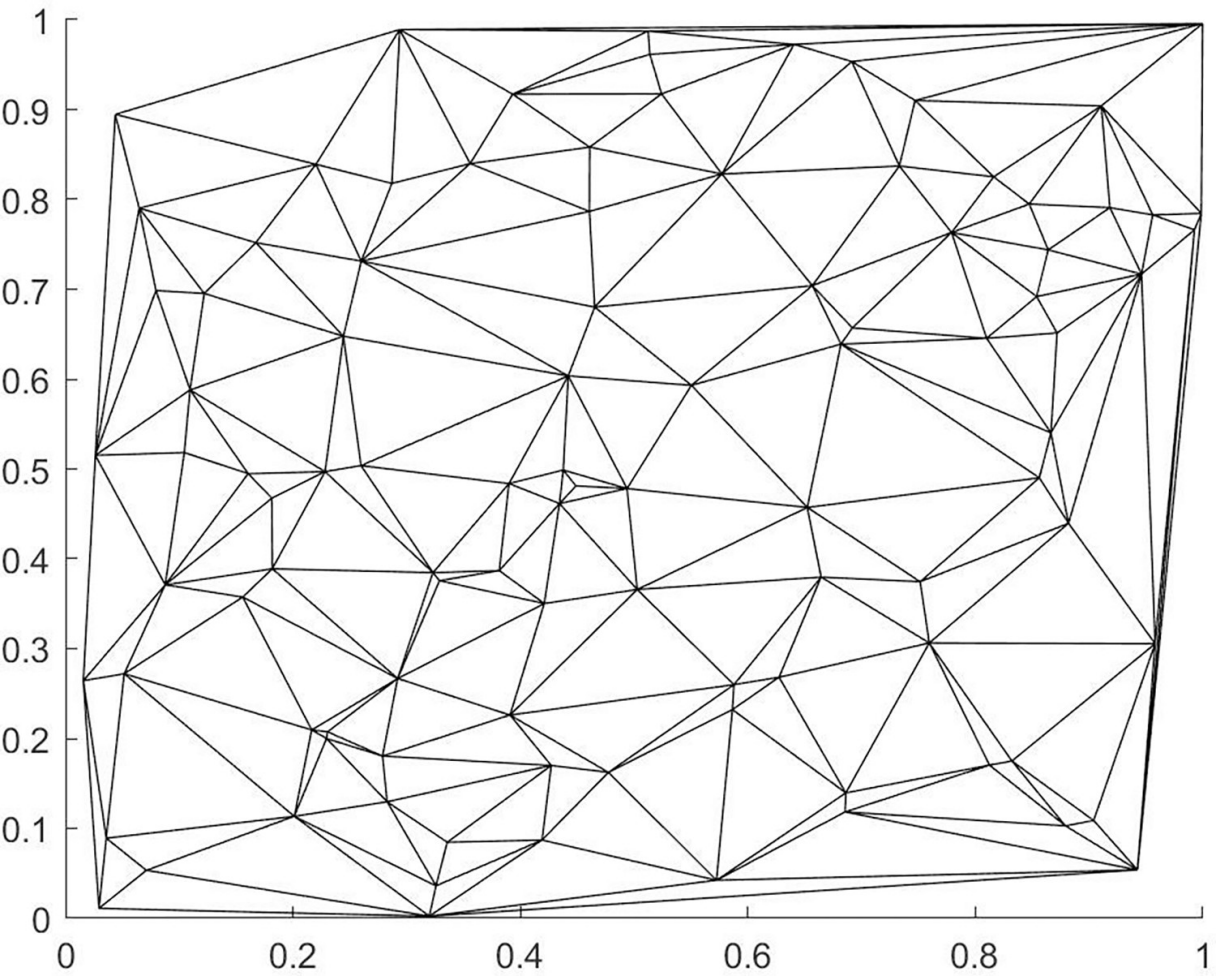
Delaunay triangulation.

The Voronoi diagram, also known as the Thiessen polygon or Dirichlet diagram, is a continuous polygon composed of the perpendicular bisectors of the lines connecting two adjacent points. The Voronoi diagram is a partitioning of the plane, characterized by the property that any point within a polygon is closer to the site of that polygon than to any site in an adjacent polygon, and each polygon contains only one site. The Voronoi diagram has the universal characteristic of dividing neighboring regions by distance and has a wide range of applications. There are many methods for generating Voronoi diagrams, including divide and conquer, scan line algorithms, and Delaunay triangulation algorithms. The characteristics of the Delaunay triangulation are as follows: 1. Empty circle property: The interior of any triangle in the Delaunay triangulation does not contain any other points, which is a criterion for creating Delaunay triangulations. 2. Maximum minimum angle property: In a triangulation of a point set, the Delaunay triangulation has the smallest minimum angle and the largest maximum angle, and the diagonal of the convex quadrilateral formed by two adjacent triangles does not increase the smallest angle after being exchanged. This paper uses an improved point-by-point insertion method to generate the Delaunay network. The construction of the Voronoi diagram consists of two steps: 1. Building the Delaunay (Delaunay) triangulation, 2. Connecting the centroids of the triangles to form lines. We demonstrate these two processes using 100 random points, and the results are shown in Figs 9 and 10 below.

After the above processes, the free surface has been divided into three types of regions, but the boundaries of these regions are not fully defined. To determine clear boundaries, this paper adopts the Voronoi diagram to define the boundaries.

The main ideas of the Delaunay triangulation are as follows:

1. Construct a super triangle that contains all scattered points and insert it into the triangle list.
2. Insert the scattered points from set S sequentially into the super triangle. Find the triangle in the triangle list that contains the insertion point within its circumcircle (called the influencing triangle of the point), delete the common edge of the influencing triangle, and connect the insertion point with all the vertices of the influencing triangle to complete the insertion of a point into the Delaunay triangle list.
3. Optimize the locally newly formed triangles according to the optimization criteria. Insert the formed triangles into the Delaunay triangle list.
4. Repeat step 2 above until all scattered points are inserted. The main steps are as follows:

The local optimization criterion in step 3 refers to optimizing the newly formed triangle, combining two triangles with common edges into polygons, and checking whether the fourth vertex is in the circumscribed circle of the triangle by using the maximum empty circle criterion. If so, the local optimization process is completed by adjusting the diagonal by exchanging the diagonal.

**Fig 11:**
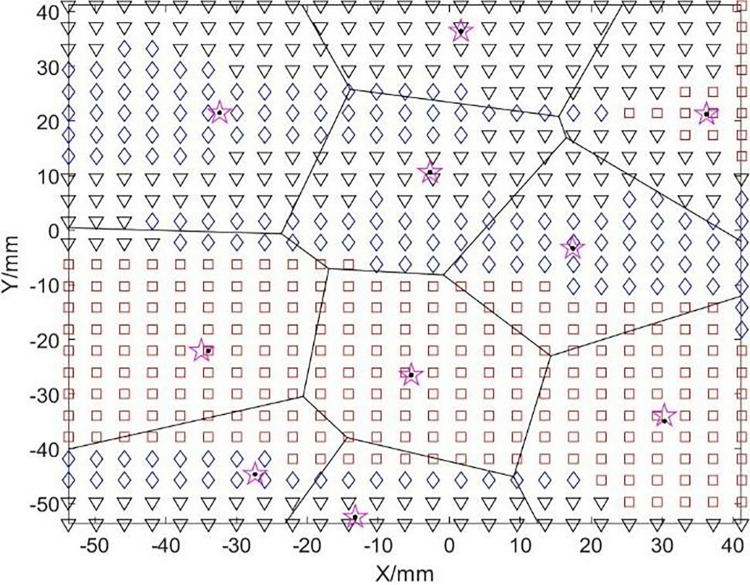
Voronoi diagram boundary division definition results.

Using the optimized cluster centers as sites, the Voronoi diagram generated defines the regional boundaries, as shown in Fig 11. From this point, the fragmentation of the free surface is complete, and the boundaries are fully defined.

## 6. Simulation experiment

To verify the effectiveness of using the fuzzy c-means method for surface segmentation in the machining process, the surface model was first constructed using UG software, and then simulated machining was performed through the three-axis milling module. According to the curvature calculation formula, the minimum radii of curvature for the concave and saddle-shaped areas on the surface were calculated to be 1.4552mm and 2.5091mm, respectively. To avoid over-cutting and wasting machining time, milling tools with diameters of 2mm and 5mm were used for machining in the concave and saddle areas, respectively. A set of traditional machining methods was designed for comparison with the method presented in this paper.

**Fig 12:**
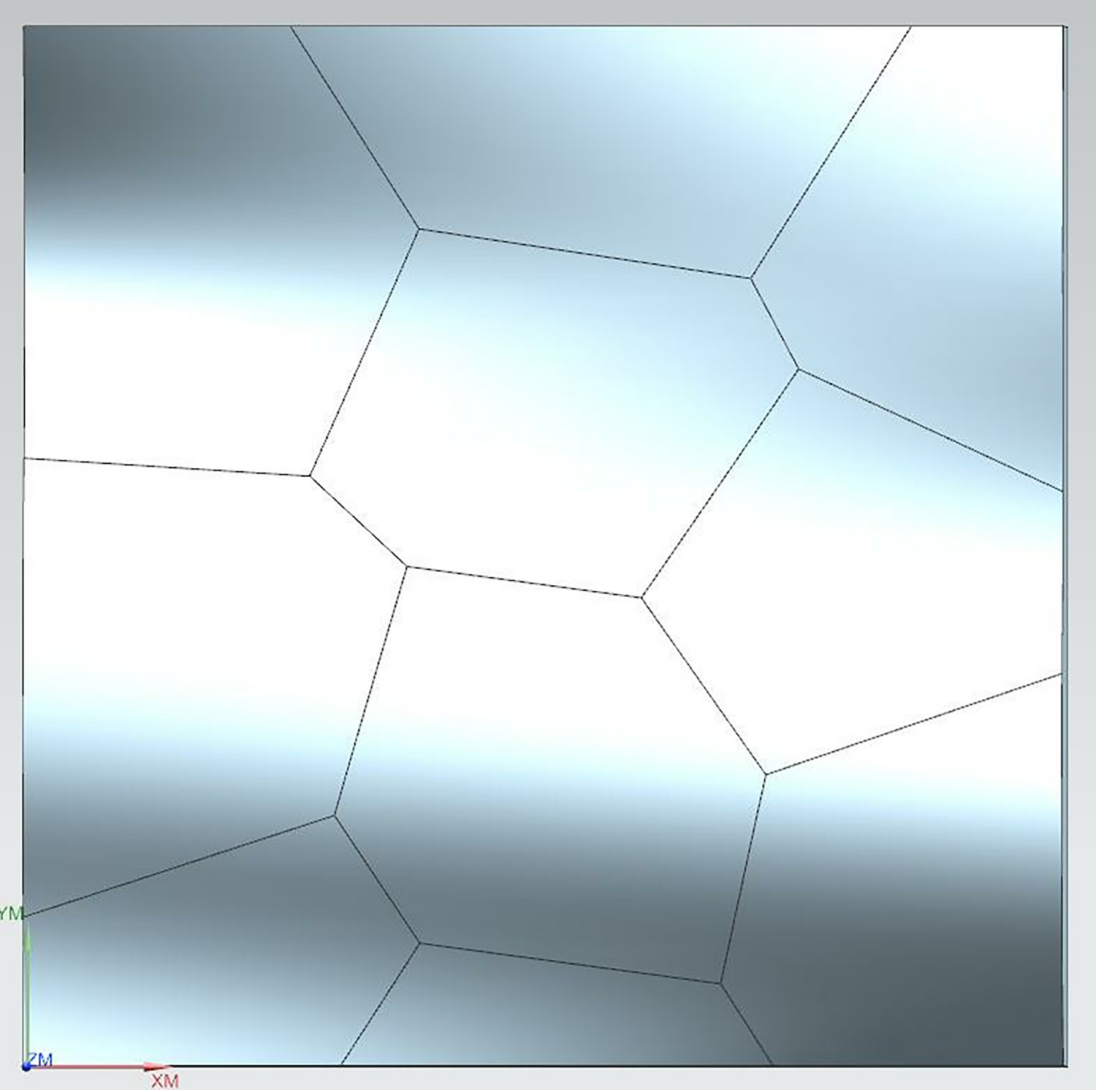
3D surface segmentation diagram.

An excessively low cutting speed can lead to low milling efficiency, while an excessively high cutting speed can easily cause increased tool wear. The suitable range of cutting speeds for processing aluminum alloy with cemented carbide tools is 100~200m/min; the suitable range of cutting depths is 0.3~2mm. The cutting speed selected in this paper is 100m/min, and based on the cutting speed, the magnitude of other machining process parameters can be calculated. The specific machining scheme is as follows:

Step 1: Generate the point cloud model of the surface using MATLAB and import the point cloud data of the surface into UG. Fit the surface using the fitting commands in UG. Construct the solid model of the workpiece by stretching the surface through a cross-section and using Boolean operations. Finally, use the projection command function to project the divided grid regions onto the surface model that requires processing. As shown in Fig 12.

**Fig 13:**
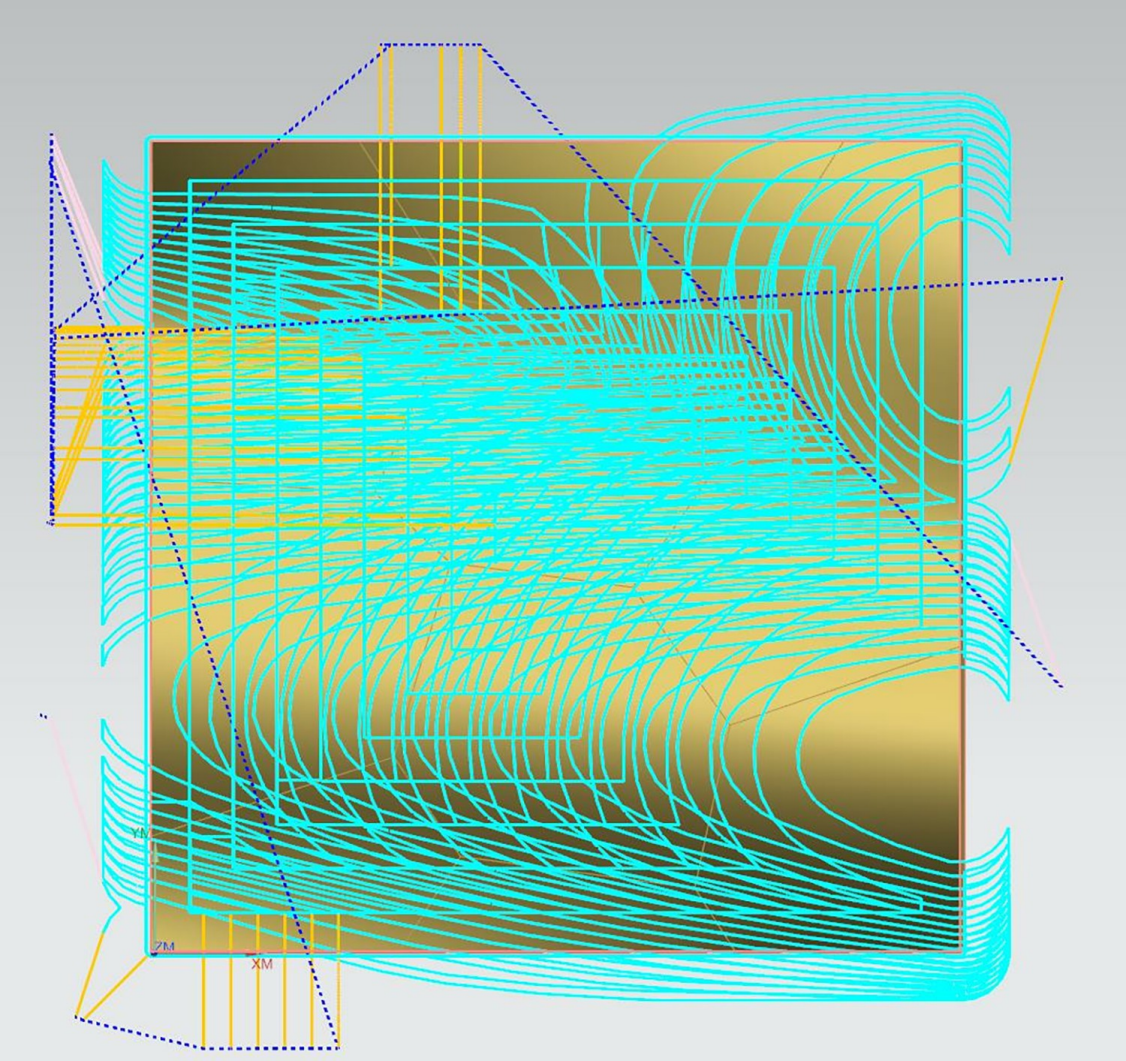
Rough machining phase.

Step 2: Using a D16 flat-end mill, the machining parameters are set as follows: spindle speed of 1990 revolutions per minute (r/min), feed rate of 1592 millimeters per minute (mm/min), and the milling method is fixed contour milling, denoted as Sample 1 and Sample 2. As shown in Fig 13.

**Fig 14:**
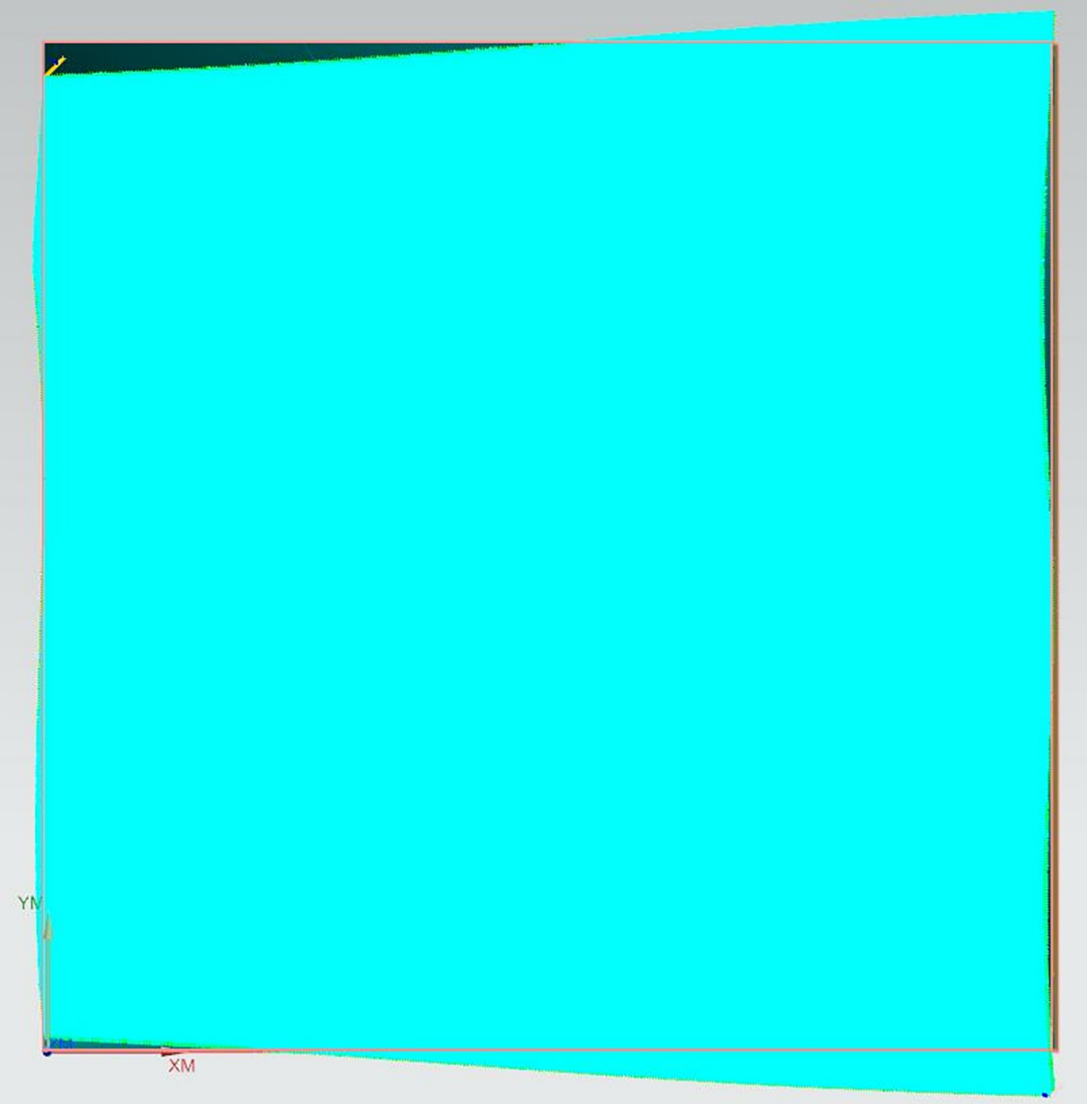
Semi-precision machining phase.

Step 3: Using a D10 ball nose cutter, the machining parameters are set as follows: spindle speed of 3180 revolutions per minute (r/min). During the semi-finishing stage, the feed rate is 1272 millimeters per minute (mm/min), with a cutting depth of 0.5mm and a residual height of 0.1mm. Samples 1 and 2 are semi-finished with these parameters. As shown in Fig 14.

**Fig 15:**
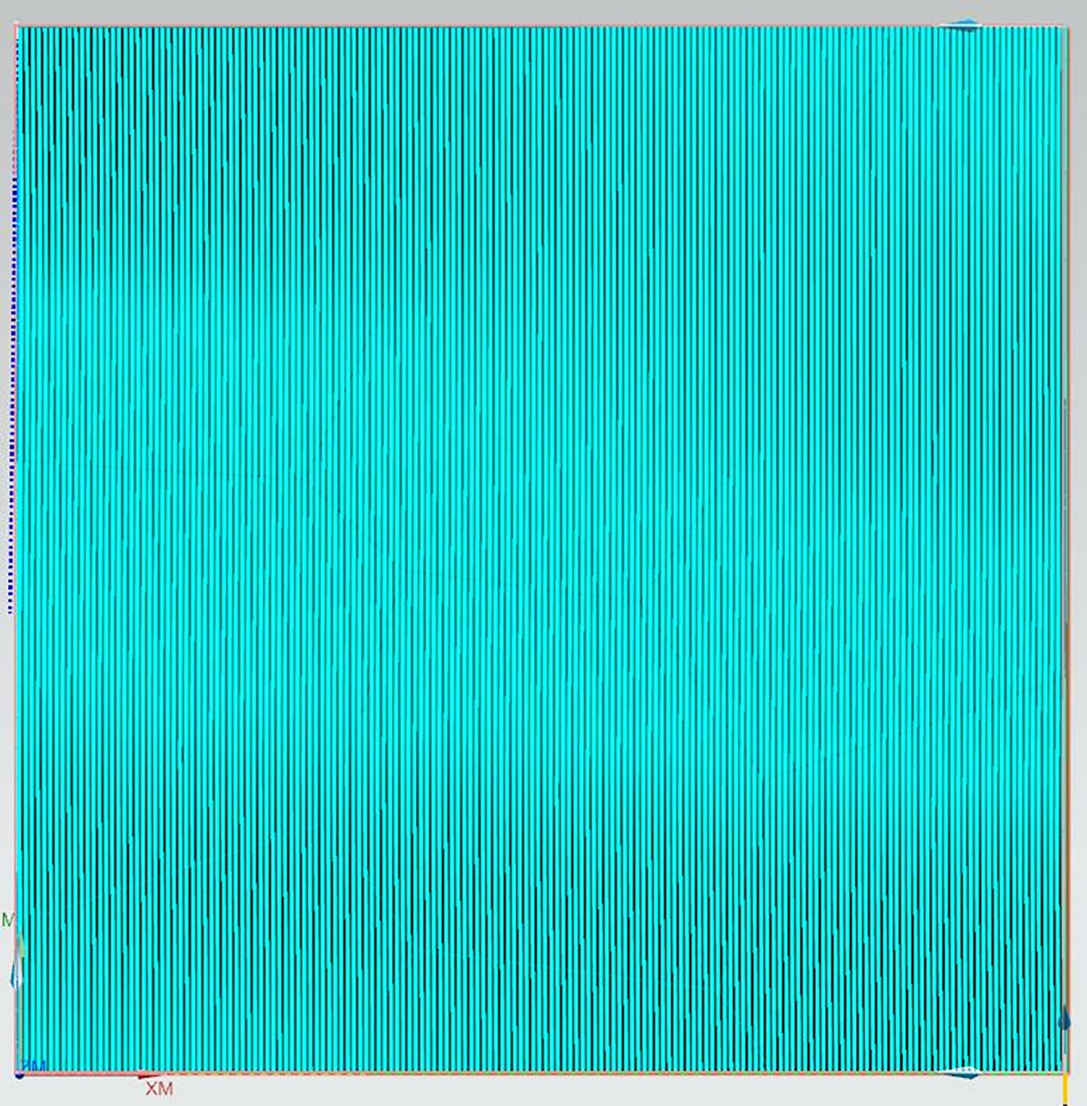
Sample 1 is subjected to overall precision machining.

**Fig 16:**
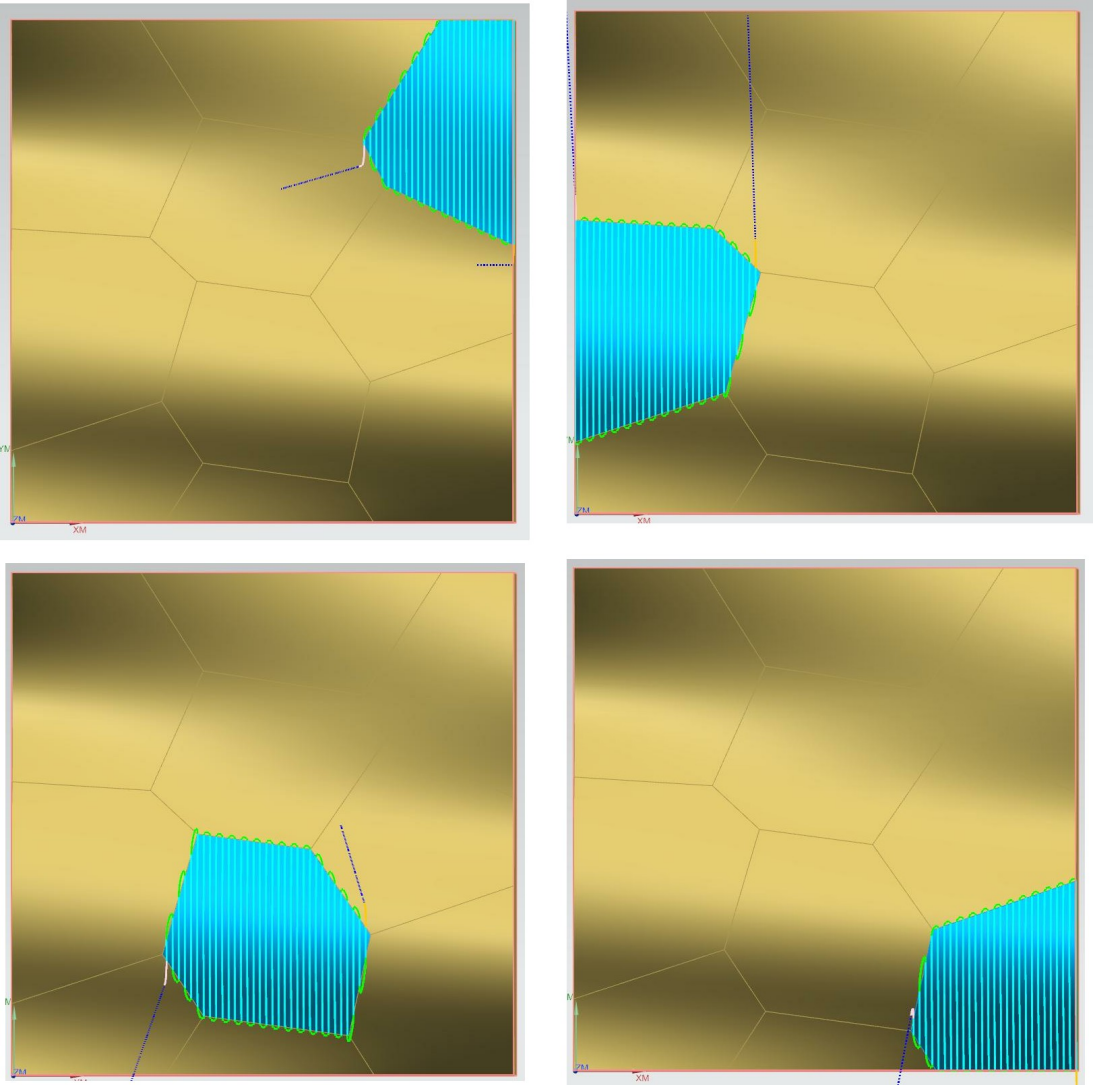
Precision machining of the convex region of Sample 2.

**Fig 17:**
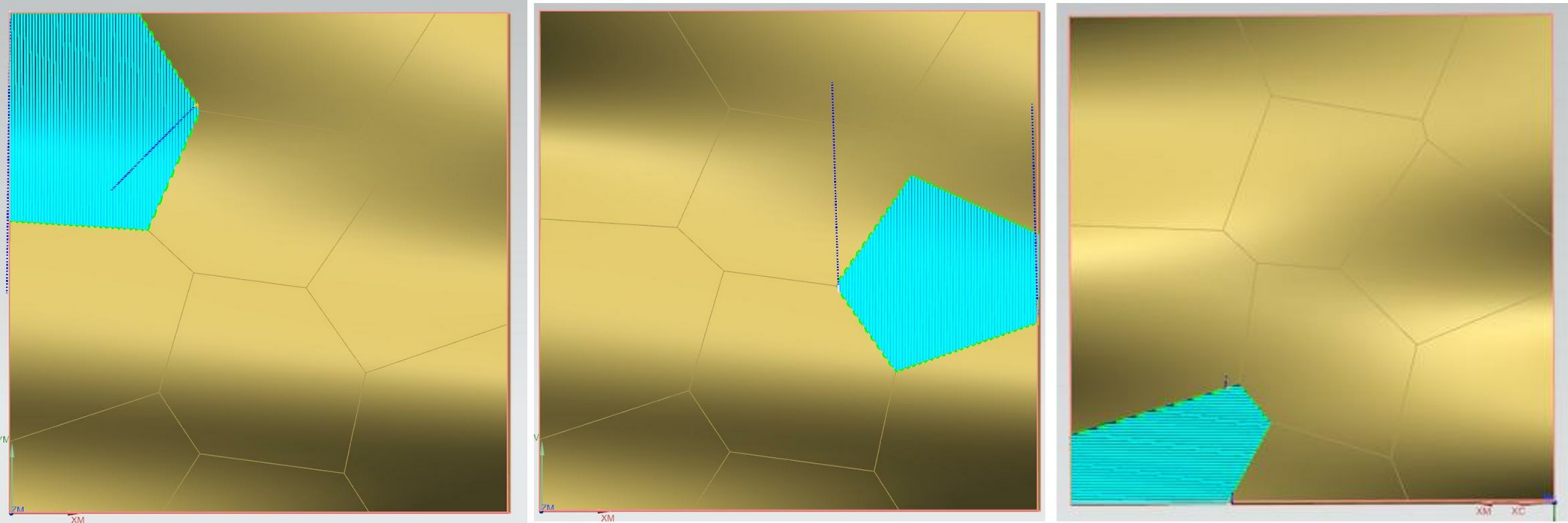
Precision machining stage of the concave region of Sample 2.

**Fig 18:**
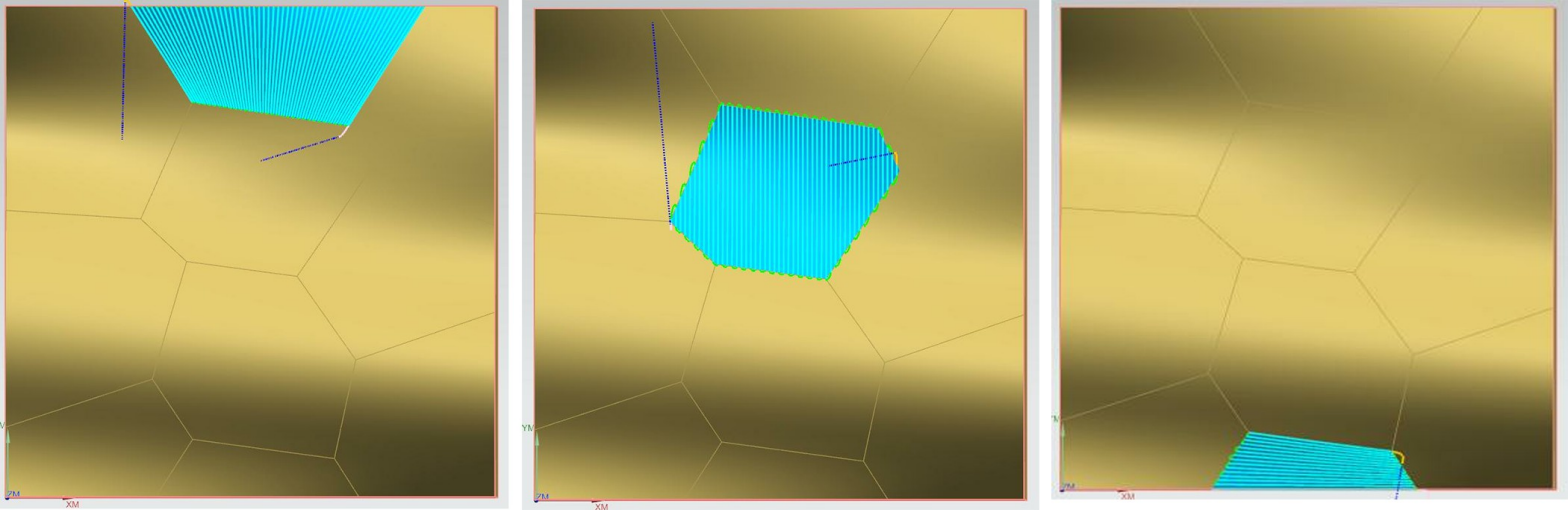
Precision machining stage of the saddle region of Sample 2.

Step 4: A 2mm diameter ball nose end mill is selected to perform overall milling on the entire surface of the curve. The spindle speed is set to 15900 revolutions per minute (r/min), the feed rate is 6360 millimeters per minute (mm/min), the cutting depth is 0.3mm, and the residual height is 0.05mm. Under the same conditions of the machining tool and machining process parameters, the method presented in this paper is applied for precision machining of different areas of Sample 2, which are specifically divided into: convex areas with a spindle speed of 4000 r/min, feed rate of 1600 mm/min, cutting depth of 0.3mm, and residual height of 0.05mm; concave areas with a spindle speed of 15900 r/min, feed rate of 6360 mm/min, cutting depth of 0.3mm, and residual height of 0.05mm; and saddle-shaped areas with a spindle speed of 6400 r/min, feed rate of 2560 mm/min, cutting depth of 0.3mm, and residual height of 0.05mm. The machining process diagrams are shown in Figs 15–18.

Academic Translation: Based on the simulation results mentioned above, combined with Table 4, it can be observed that the segmented processing method has reduced the machining path by 12.18%, and has also decreased the processing time by 13.93%.

**Table 4:** Comparison of simulation results.

Simulation results Processing method	Toolpath length /(mm)	Processing time /(s)
Traditional method	17437	359
Proposed method	15314	309

## 7. Experimental verification of surface partitioning

Academic Translation: Whether the surface partitioning is reasonable, whether the simulation of the CNC machining path is correct, whether the length of the machining path is reduced, and whether the machining efficiency is high all need to be verified through experiments. Therefore, this section uses a three-axis CNC milling machine as the experimental platform, and conducts comparative experiments between the different partitioning methods and machining processes for the same processing surface and the traditional overall processing method. This is done to verify the above issues.

### 7.1 Experimental conditions

**Fig 19:**
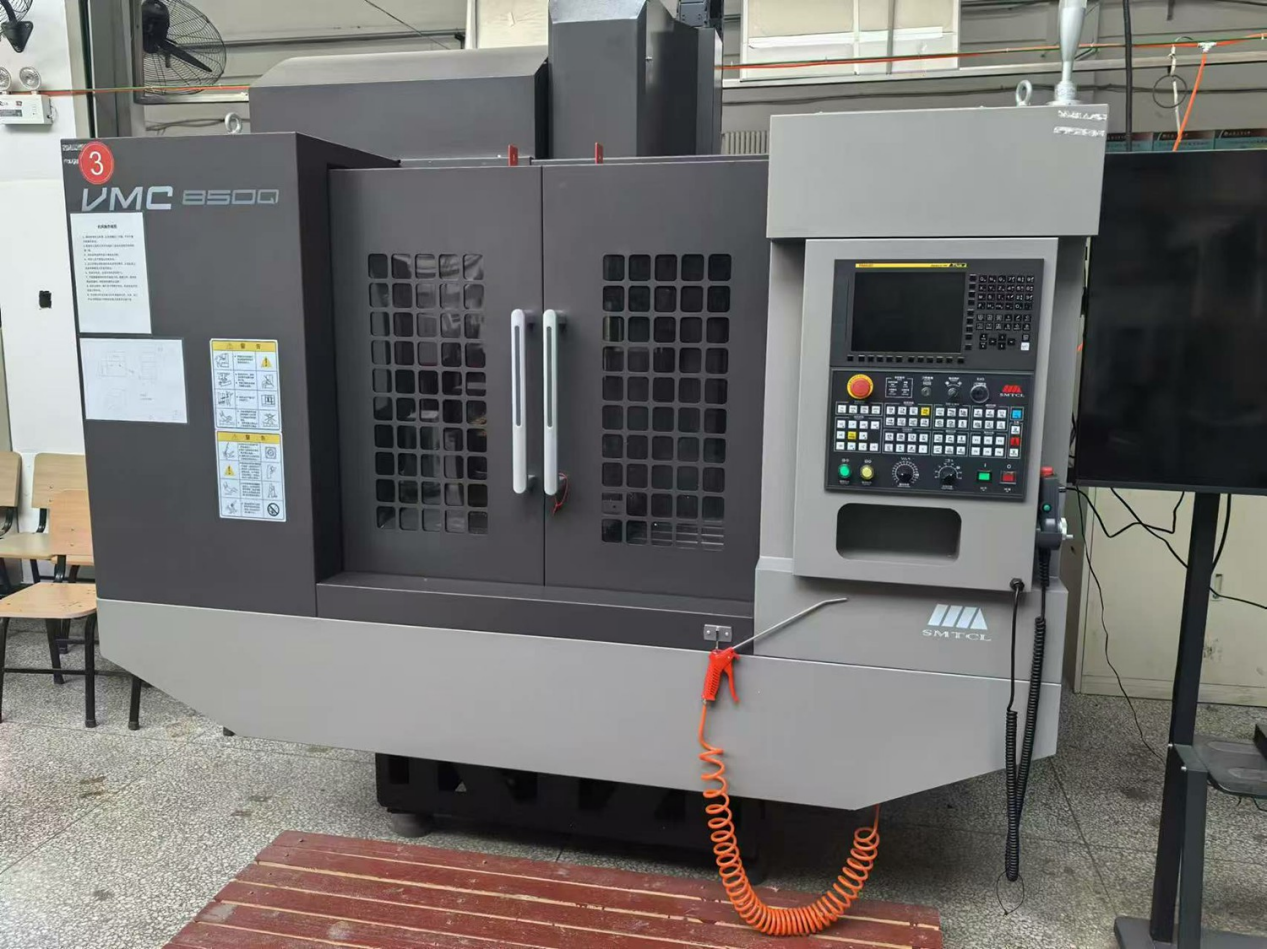
Vertical machining center VMC850.

**Fig 20:**
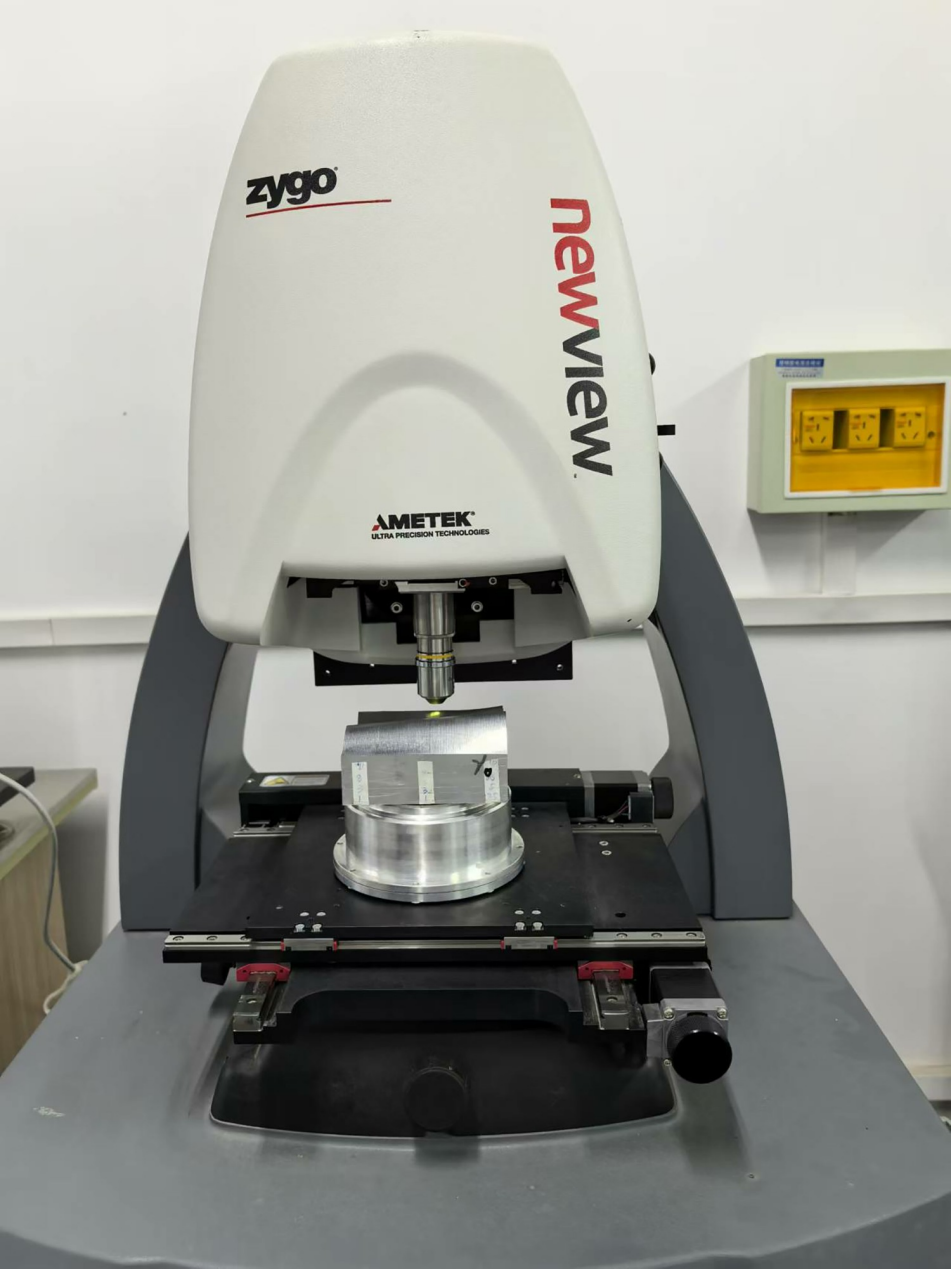
Zygo white light interferometer.

Experimental Equipment: The machining center is a vertical machining center VMC850Q, and the measuring instrument is a Zygo white light interferometer. As shown in Figs 19 and 20.

Experimental Material: 6061 aluminum alloy, with a workpiece machining size of 10mm × 10mm.

Machining Tools: Integral cemented carbide flat-end mill and double-edged ball nose mill.

**Fig 21:**
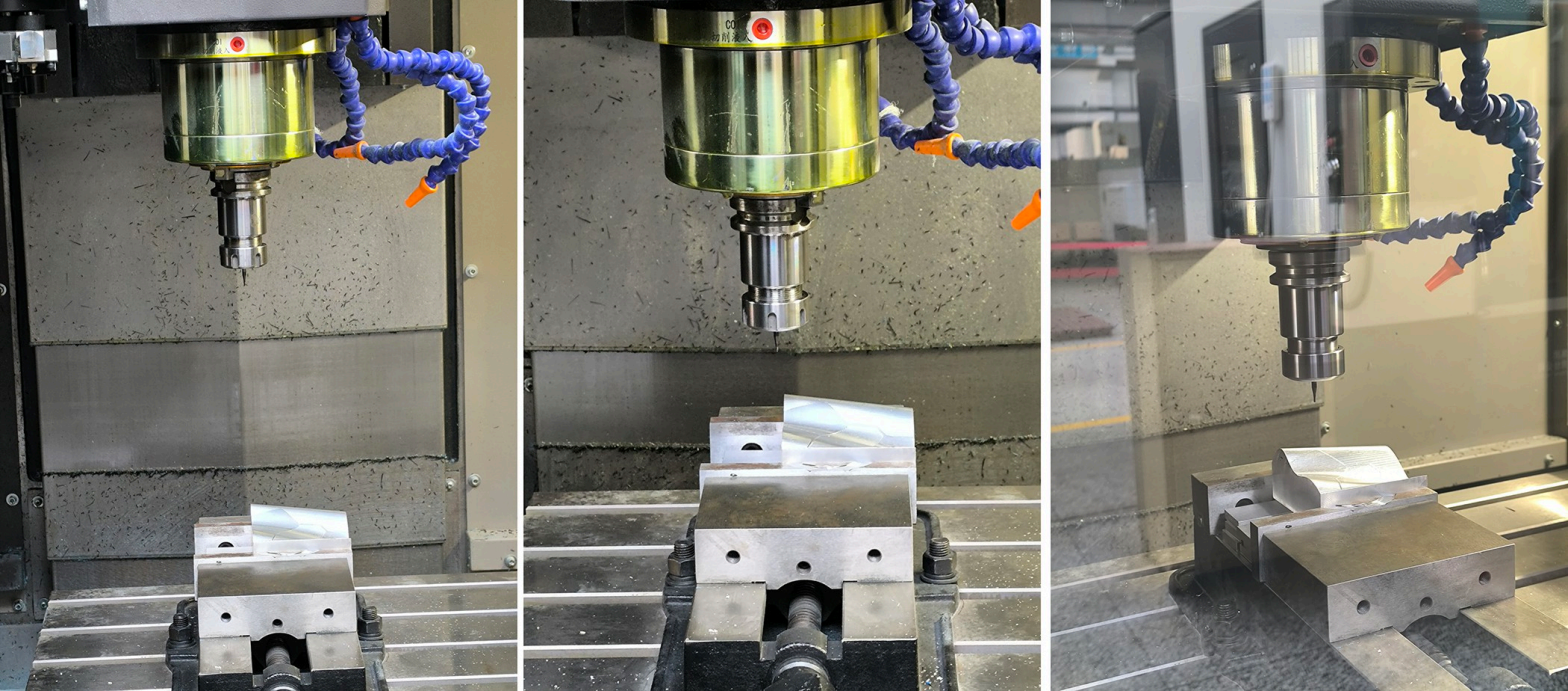
Machining process diagram.

The processing process is shown in Fig 21.

The machining parameters for each stage are shown in Table 5.

**Table 5:** Table of machining parameters for each stage.

Operation Process	Cutting Area	Tool Size (mm)	Spindle Speed (r/min)	Feed Rate (mm/min)	Residual Height (mm)	Cutting Depth (mm)
Rough Machining	Entire Surface	D16	1990	1592	−	1
Semi-Finish Machining	Entire Surface	D10	3180	1272	0.1	0.5
Finish Machining	Convex Area	D8	4000	1600	0.05	0.3
	Saddle Area	D5	6400	2560	0.05	0.3
	Concave Area	D2	15900	6360	0.05	0.3
	Entire Area	D2	15900	6360	0.05	0.3

### 7.2 Experimental results and analysis

The comparative experimental results are shown in Table 6.

**Table 6:** Comparison of experimental results.

Simulation results Processing method	Toolpath length /(mm)	Processing time /(s)
Traditional method	21799	462
Proposed method	19123	404

**Fig 22:**
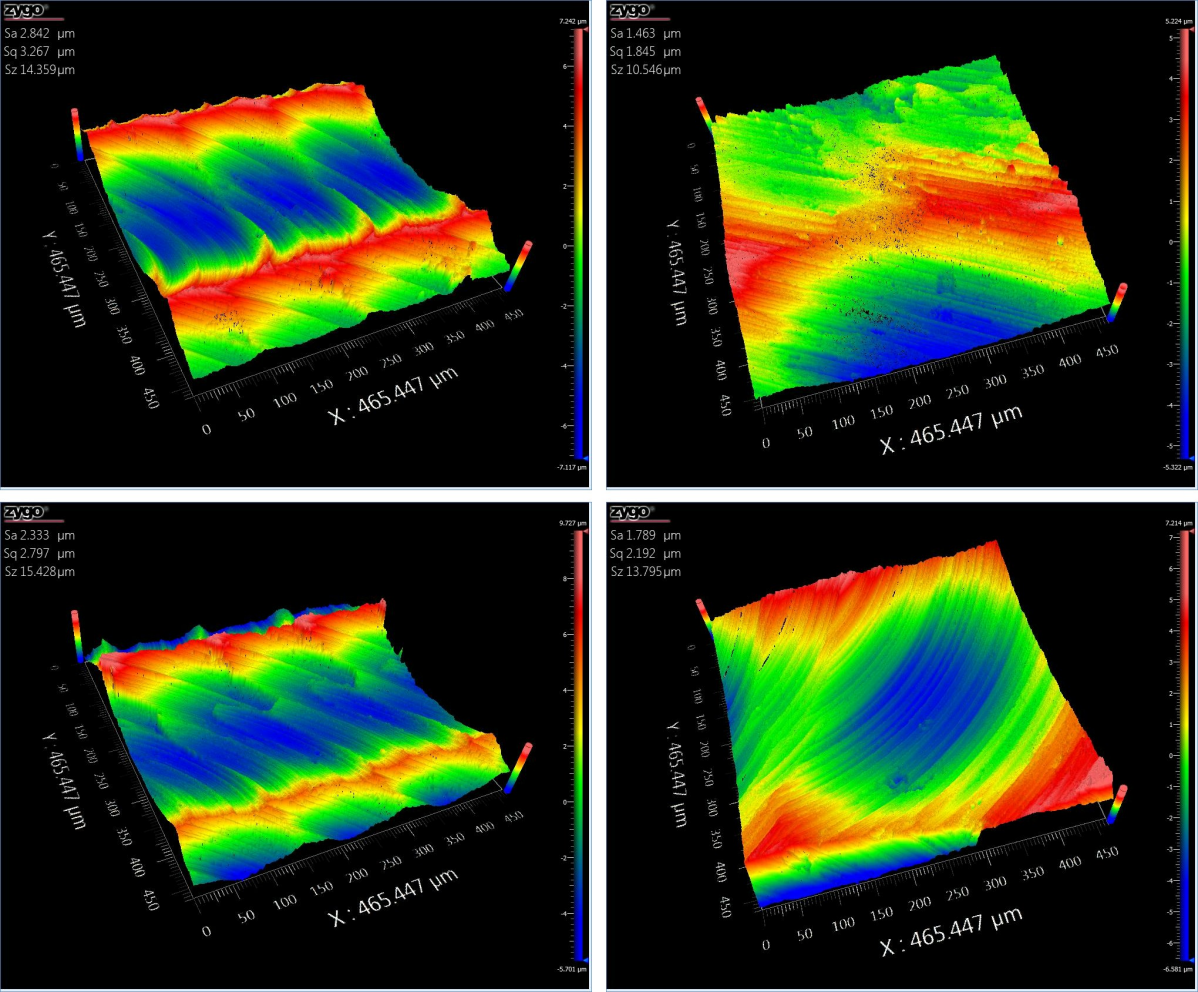
Academic translation: Comparative figure of convex area processing with and without segmentation. (a) Three-dimensional topography map of the unsegmented convex area 2. (b) Three-dimensional topography map of the segmented convex area 2. (c) Three-dimensional topography map of the unsegmented convex area 4. (d) Three-dimensional topography map of the segmented convex area 4.

**Fig 23:**
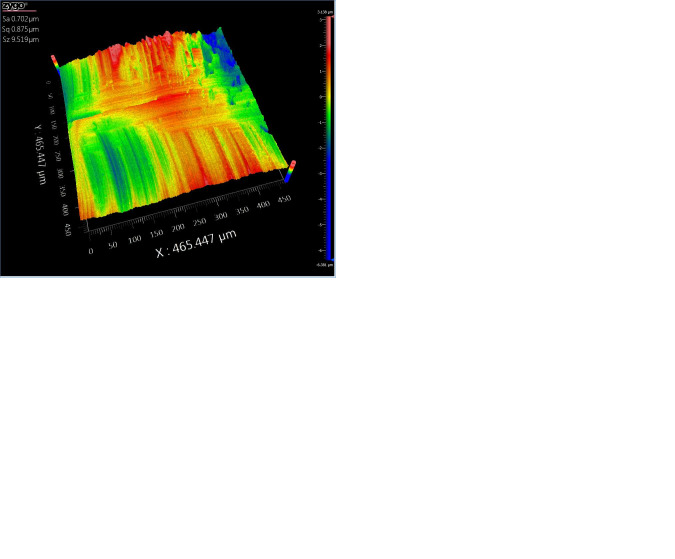
Comparative figure of concave area processing with and without segmentation. (a)Three-dimensional topography map of the unsegmented concave area 5. (b) Three-dimensional topography map of the segmented concave area 5. (c) Three-dimensional topography map of the unsegmented concave area 6. (d) Three-dimensional topography map of the segmented concave area 6.

**Fig 24:**
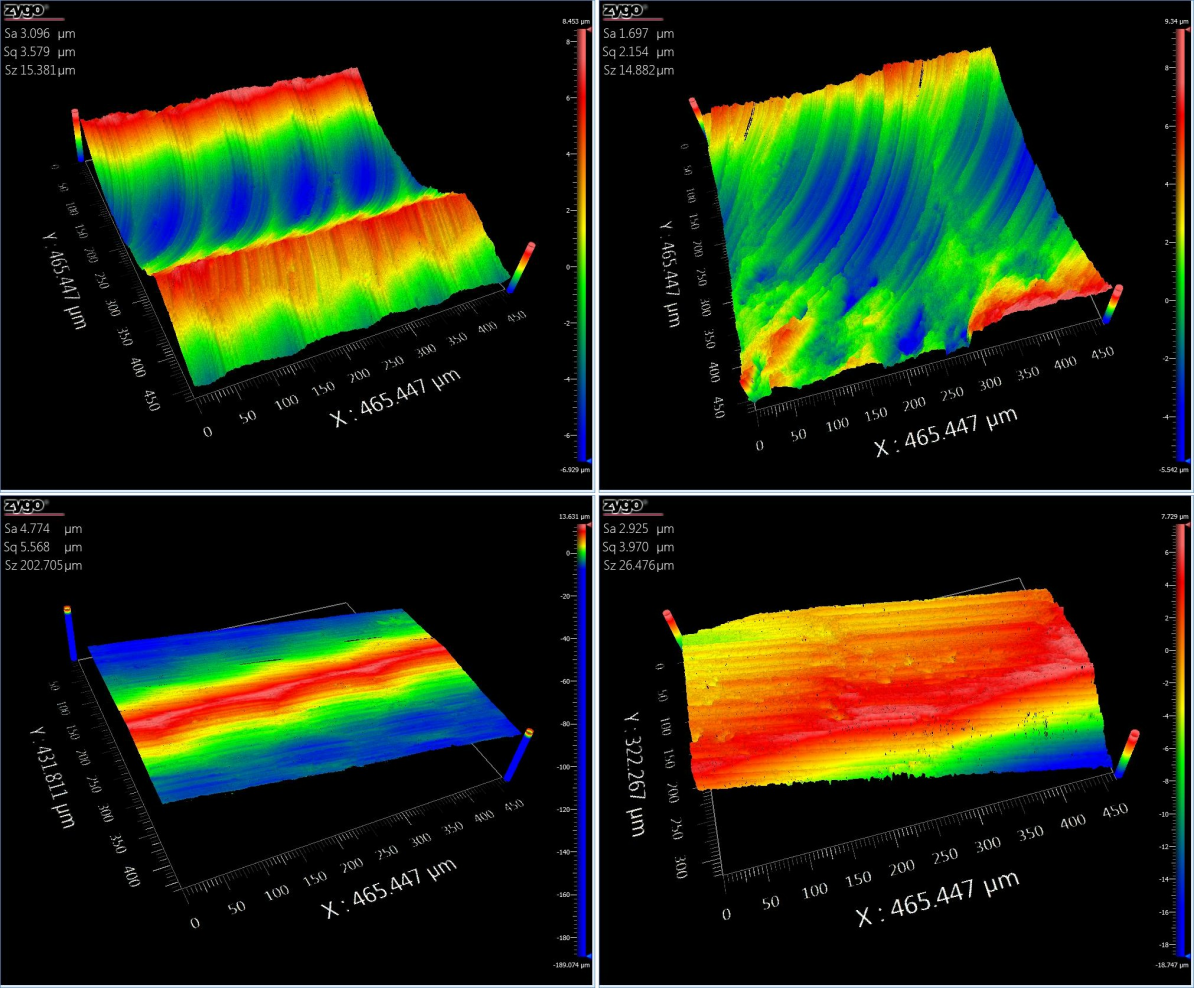
Academic translation: Comparative figure of saddle area processing with and without segmentation. (a)Three-dimensional topography map of the unsegmented saddle area 9. (b) Three-dimensional topography map of the segmented saddle area 9. (c) Three-dimensional topography map of the unsegmented saddle area 10. (d)Three-dimensional topography map of the segmented saddle area 10.

The comparison of the three-dimensional surface topography is shown in Figs 22–24.

Based on the results from Table 4 and Figs 22–24, it can be observed that the segmented processing method has reduced the machining path by 12.28% and the machining time by 12.56%. The surface roughness of the unsegmented convex areas ranged from 2.333μm to 2.842μm, while the surface roughness of the segmented convex areas ranged from 1.463μm to 1.789μm. The surface roughness of the unsegmented concave areas ranged from 0.878μm to 1.554μm, while the surface roughness of the segmented concave areas ranged from 0.702μm to 1.243μm. The surface roughness of the unsegmented saddle areas ranged from 3.096μm to 4.774μm, while the surface roughness of the segmented saddle areas ranged from 1.697μm to 2.925μm. This indicates that the segmented processing method, compared to traditional processing methods, not only reduces the machining path and time, effectively improving machining efficiency, but also enhances the surface quality.

## 8. Conclusion

To address the issues of poor surface quality and low efficiency in the machining of complex surfaces, by analyzing the intrinsic properties of the surface, a free-form surface model is defined using NURBS, this study combines the fuzzy c-means clustering algorithm with the Voronoi diagram algorithm to divide free surfaces and define boundaries, and selects different methods for processing the divided surfaces.

1. Through a comparative simulation of machining with the same profile but different machining processes, the traditional machining method and the segmented machining method are analyzed and compared. It is found that the segmented machining method of free surfaces reduces the machining path by 12.18% and the machining time by 13.93%, effectively improving machining efficiency.
2. Experimental results indicate that the segmented processing method has reduced the machining path length by 12.28% and the machining time by 12.56%, demonstrating that compared to the integral processing method, the segmented processing method can effectively enhance the efficiency and quality of free-form surface machining. The results also validate the accuracy of the simulation processing.
